# Supplementary material for: Individual retrotransposon integrants are differentially controlled by KZFP/KAP1-dependent histone methylation, DNA methylation and TET-mediated hydroxymethylation in naïve embryonic stem cells
Source: Epigenetics Chromatin. 2018 Feb 26;11:7. doi: 10.1186/s13072-018-0177-1 (PMC6389204; doi:10.1186/s13072-018-0177-1)
Supplement: Supplementary file 11 — Additional file 11. Pattern analysis. [file 13072_2018_177_MOESM11_ESM.zip › Patterns analysis/DataTables/extensions/Responsive/examples/display-control/classes.html]

Responsive example - Class control


# Responsive example Class control

You can tell Responsive what columns to want to be visible on different devices through the use of
class names on the columns. The breakpoints are horizontal screen resolutions and the defaults are set
for common devices:

- `desktop` x >= 1024px
- `tablet-l` (landscape) 768 <= x < 1024
- `tablet-p` (portrait) 480 <= x < 768
- `mobile-l` (landscape) 320 <= x < 480
- `mobile-p` (portrait) x < 320

You may leave the `-[lp]` option from the end if you wish to just target all tablet or
mobile devices. Additionally to may add `min-`, `max-` or `not-` as a
prefix to the class name to perform logic operations. For example `not-mobile` would cause a
column to appear as visible on desktop and tablet devices, while `min-tablet-l` would
require at least a horizontal width of 768 for the browser window to be shown, and be shown at all
sizes larger.

Additionally, there are three special class names:

- `all` - Always display
- `none` - Don't display as a column, but show in the child row
- `never` - Never display
- `control` - Used for the `column` `responsive.details.typeR`
  option.

Please refer to the Responsive manual for
further details of these options.

This example shows the `salary` column visible on a desktop only - `office`
and `age` require a tablet, while the `position` column requires a phone in
landscape or larger. The `name` column is always visible and the `start date` is
never visible.

This can be useful if you wish to change the format of the data shown on different devices, for
example using a combination of `mobile` and `not-mobile` on two different columns
would allow information to be formatted suitable for each device type.

| Name | Position | Office | Age | Start date | Salary | Extn. |
| --- | --- | --- | --- | --- | --- | --- |
| Name | Position | Office | Age | Start date | Salary | Extn. |
| --- | --- | --- | --- | --- | --- | --- |

- Javascript
- HTML
- CSS
- Ajax
- Server-side script

The Javascript shown below is used to initialise the table shown in this
example:

`$(document).ready(function() {
$('#example').DataTable( {
"ajax": "../../../../examples/ajax/data/objects.txt",
"columns": [
{ "data": "name" },
{ "data": "position" },
{ "data": "office" },
{ "data": "extn" },
{ "data": "start_date" },
{ "data": "salary" },
{ "data": "extn" }
]
} );
} );`

In addition to the above code, the following Javascript library files are loaded for use in this
example:

- ../../../../media/js/jquery.js
- ../../../../media/js/jquery.dataTables.js
- ../../js/dataTables.responsive.js

The HTML shown below is the raw HTML table element, before it has been enhanced by
DataTables:

This example uses a little bit of additional CSS beyond what is loaded from the library
files (below), in order to correctly display the table. The additional CSS used is shown
below:

The following CSS library files are loaded for use in this example to provide the styling of the
table:

- ../../../../media/css/jquery.dataTables.css
- ../../css/dataTables.responsive.css

This table loads data by Ajax. The latest data that has been loaded is shown below. This data
will update automatically as any additional data is loaded.

The script used to perform the server-side processing for this table is shown below. Please note
that this is just an example script using PHP. Server-side processing scripts can be written in any
language, using the protocol described in the
DataTables documentation.

## Other examples

### Basic initialisation

- Class name
- Configuration option
- `new` constructor
- Ajax data
- Default initialisation

### Styling

- Bootstrap styling
- Foundation styling

### Display control

- Automatic column hiding
- Class control
- Assigned class control

### Child rows

- Disable child rows
- Column controlled child rows
- Column control - right
- Whole row child row control
- Custom child row renderer

Please refer to the DataTables documentation for full
information about its API properties and methods.  
Additionally, there are a wide range of extras and
plug-ins which extend the capabilities of
DataTables.

DataTables designed and created by SpryMedia Ltd © 2007-2014  
DataTables is licensed under the MIT license.
